# Editorial: Quantitative [^18^F]NaF PET in metastatic and metabolic bone diseases

**DOI:** 10.3389/fnume.2023.1343913

**Published:** 2023-12-11

**Authors:** Tanuj Puri, Amelia E. Moore, Abhishek Mahajan, Alan McWilliam, Marie H. Vrist, Glen M. Blake

**Affiliations:** ^1^ Faculty of Biology, Medicine and Health, School of Medical Sciences, Division of Cancer Sciences, The University of Manchester, The Christie NHS Foundation Trust, Manchester, United Kingdom; ^2^ Department of Cancer Imaging, School of Biomedical Engineering and Imaging Sciences, King’s College London, St. Thomas’ Hospital, London, United Kingdom; ^3^ The Clatterbridge Cancer Centre NHS Foundation Trust, University of Liverpool, Liverpool, United Kingdom; ^4^ Gødstrup Hospital, University Clinic in Nephrology and Hypertension, Herning, Denmark

**Keywords:** [^18^F] sodium fluoride PET-CT, [^18^F]NaF PET-CT, quantitative measurement of bone, metastatic bone disease, metabolic bone disease, SUV, K_i_

**Editorial on the Research Topic** Quantitative [^18^F]NaF PET in metastatic and metabolic bone diseases

Quantitative sodium fluoride positron emission tomography - computed tomography ([^18^F]NaF PET-CT) has emerged as a valuable research tool for assessing bone metabolism. It provides a non-invasive means to quantify bone remodeling, serving as a surrogate measure for local bone formation rates. The method has been validated against the gold standard of bone biopsy, which is an invasive, uncomfortable, and painful technique for patients. Biochemical markers of bone formation and resorption in serum or urine provide a rapid and simple way of measuring whole-body skeletal metabolism, which can be used to verify treatment response within a few weeks after the commencement of treatment. However, unlike imaging methods such as [^18^F]NaF PET-CT, they are unable to provide site-specific information at clinically important sites susceptible to fractures, such as the hip and spine.

[^18^F]NaF PET has proven useful in early-phase clinical trials of novel anti-fracture drugs, helping to mitigate late attrition rates. The articles published within this Research Topic delve into the nuances of [^18^F]NaF PET, exploring its applications in metastatic and metabolic bone diseases. These articles not only highlight current advancements in the field but also set the stage for future directions. Here, we summarise insights from four articles that contribute to the expanding landscape of knowledge in this field.

In an introductory review article, Puri et al. underscore the capacity of [^18^F]NaF PET to noninvasively estimate changes in regional bone metabolism far earlier than the structural alterations detected by conventional methods such as dual-energy x-ray absorptiometry (DXA). The review discusses the two commonly used approaches to applying [^18^F]NaF PET to study bone metabolism: the measurement of [^18^F]NaF bone plasma clearance (K_i_) and the standardised uptake value (SUV). Crucially, it highlights instances where a decoupling of the correlation between SUV and K_i_ may occur, emphasising the greater reliability of K_i_ measurement when a bone lesion or its treatment is potent enough to alter the area under the arterial input function curve (AUC) compared to that observed prior to treatment. Furthermore, the article discusses advancements in PET data methodologies, such as shortened scan times and a reduced number of blood samples, enhancing the practicality of [^18^F]NaF PET outside the research setting and fostering its adoption for a wider variety of clinical applications.

In the second article by Theil et al., the focus shifts to the meticulous evaluation of image-derived input functions in patients with chronic kidney disease mineral and bone disorder (CKD-MBD). The article systematically analyses and compares multiplicative and additive methods of deriving semi-population arterial input functions, suggesting that the resulting AUCs are within 12% of each other and yield similar K_i_ values. Furthermore, the article suggests that input function recovery coefficients correlate with the volume and location of the region within the artery from which these values are derived. Finally, the article recommends the use of a consistent methodology for deriving the input function to minimise the variation in the assessment of bone metabolism between diverse patient population studies and to improve the differentiation between states of low and high bone turnover rates within the CKD-MBD population.

The third article by Hardcastle et al. contributes clinical results, concentrating on the utility of [^18^F]NaF PET-CT in assessing the response to stereotactic ablative body radiation therapy (SABR) in breast cancer bone metastases. The findings underscore the ability of [^18^F]NaF PET to quantify changes in SUV_max_ and SUV_mean_ post-SABR. The study recommends further research to explore the use of [^18^F]NaF PET in conjunction with other imaging modalities to more accurately assess treatment response in oligometastatic disease.

In the fourth article, Watkins et al. explore the impact of dynamic [^18^F]NaF PET scan duration on kinetic uptake parameters in the knee with and without osteoarthritis. Addressing practical concerns related to patient comfort and scan throughput, the study demonstrates the feasibility of shorter scan times while retaining accuracy in estimating bone perfusion and metabolism. This finding holds implications for improving the efficiency of PET scans, reducing patient burden, and enhancing overall clinical workflow.

**Figure 1:**
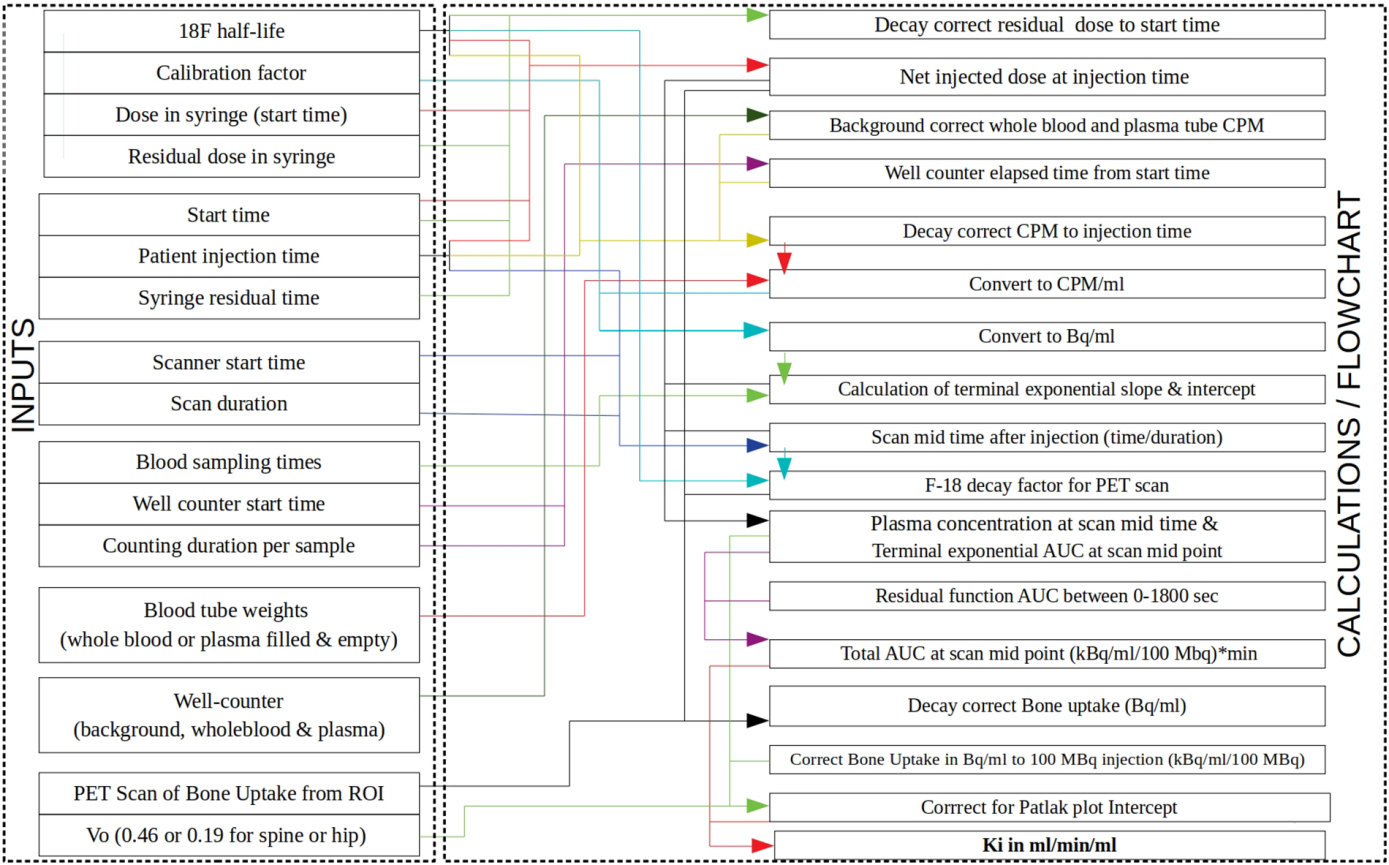
Block diagram showing how the Excel spreadsheet provided in Puri et al. (1) calculates [^18^F]NaF bone plasma clearance (K_i_) values. The left-hand side column within the Excel spreadsheet lists the inputs (activities, times, well-counter counts, bone uptake, etc.) with arrows showing how they are used in the calculations shown on the right-hand side column. PET, positron emission tomography; ROI, region of interest; CPM, counts per minute; AUC, area under the curve.

As we navigate through these articles, a comprehensive understanding of the potential applications of [^18^F]NaF PET in bone diseases emerges. A frequent theme that emerges from this Research Topic is that a standardised methodology is required for deriving the input function and the corresponding K_i_ values before the technique of quantitative [^18^F]NaF PET can be translated into the clinic. For this reason, we have previously developed and shared a simplified method using a static 4-min [^18^F]NaF PET-CT scan at a single bed-position with 2–3 venous blood samples (Figure 1) and provided a spreadsheet for calculating the bone plasma clearance (K_i_) values (1).

In conclusion, the collection of articles presented in this Frontiers Research Topic underscores the transformative potential of [^18^F]NaF PET in the realm of bone diseases. From refining quantitative methodologies to assessing treatment responses, these studies contribute significantly to the evolving landscape of applying [^18^F]NaF PET imaging to estimate bone metabolism. As research continues to unfold, the integration of [^18^F]NaF PET into routine clinical practice will help revolutionise our approach to diagnosing and monitoring the treatment response in bone diseases, ultimately improving patient outcomes.
